# Nitrogen-Doped Sponge Ni Fibers as Highly Efficient Electrocatalysts for Oxygen Evolution Reaction

**DOI:** 10.1007/s40820-019-0253-5

**Published:** 2019-03-09

**Authors:** Kaili Zhang, Xinhui Xia, Shengjue Deng, Yu Zhong, Dong Xie, Guoxiang Pan, Jianbo Wu, Qi Liu, Xiuli Wang, Jiangping Tu

**Affiliations:** 1 State Key Laboratory of Silicon Materials, Key Laboratory of Advanced Materials and Applications for Batteries of Zhejiang Province, Department of Materials Science and Engineering, Zhejiang University, Hangzhou, 310027 People’s Republic of China; 2 Guangdong Engineering and Technology Research Center for Advanced Nanomaterials, School of Environment and Civil Engineering, Dongguan University of Technology, Dongguan, 523808 People’s Republic of China; 3 Department of Materials Chemistry, Huzhou University, Huzhou, 313000 People’s Republic of China; 4 Zhejiang Provincial Key Laboratory for Cutting Tools, Taizhou University, Taizhou, 318000 People’s Republic of China; 5 Department of Physics, City University of Hong Kong, Kowloon, 999077 Hong Kong; 6 Key Laboratory of Advanced Energy Materials Chemistry (Ministry of Education), College of Chemistry, Nankai University, Tianjin, 300071 China

**Keywords:** Oxygen evolution reaction, Electrocatalysis, Nickel, Sponge Structure, Electrochemical energy conversion

## Abstract

Controllable synthesis of highly active micro/nanostructured metal electrocatalysts for oxygen evolution reaction (OER) is a particularly significant and challenging target. Herein, we report a 3D porous sponge-like Ni material, prepared by a facile hydrothermal method and consisting of cross-linked micro/nanofibers, as an integrated binder-free OER electrocatalyst. To further enhance the electrocatalytic performance, an N-doping strategy is applied to obtain N-doped sponge Ni (N-SN) for the first time, via NH_3_ annealing. Due to the combination of the unique conductive sponge structure and N doping, the as-obtained N-SN material shows improved conductivity and a higher number of active sites, resulting in enhanced OER performance and excellent stability. Remarkably, N-SN exhibits a low overpotential of 365 mV at 100 mA cm^−2^ and an extremely small Tafel slope of 33 mV dec^−1^, as well as superior long-term stability, outperforming unmodified sponge Ni. Importantly, the combination of X-ray photoelectron spectroscopy and near-edge X-ray adsorption fine structure analyses shows that γ-NiOOH is the surface-active phase for OER. Therefore, the combination of conductive sponge structure and N-doping modification opens a new avenue for fabricating new types of high-performance electrodes with application in electrochemical energy conversion devices.

**Electronic supplementary material:**

The online version of this article (10.1007/s40820-019-0253-5) contains supplementary material, which is available to authorized users.

## Highlights

- Freestanding N-doped sponge Ni micro/nanofibers exhibit a porous sponge structure.
- An N-doping strategy is adopted to optimize the catalytic activity.
- γ-NiOOH is identified as active phase by XPS and NEXAFS analyses.

**Electronic supplementary material:**

The online version of this article (10.1007/s40820-019-0253-5) contains supplementary material, which is available to authorized users.

## Graphical abstract

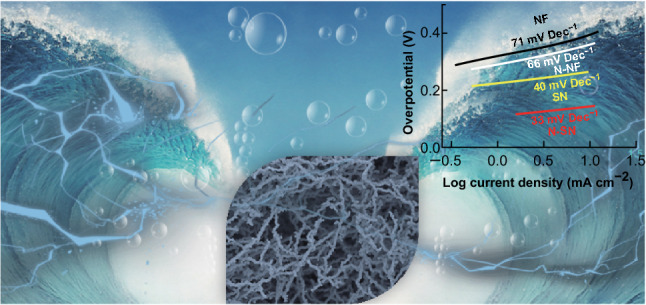

## Introduction

Owing to the huge concerns associated with serious environmental pollution and rapid fossil energy consumption, the development of clean and renewable energy technologies has become a vital task [1–4]. The oxygen evolution reaction (OER), as a key process in water splitting and rechargeable metal-air batteries, has attracted considerable attention for decades [5–14]. However, the OER is a kinetically sluggish process with a high overpotential, which calls for efficient electrocatalysts that can reduce the overpotential and improve the reaction efficiency [15–19]. Currently, noble metal oxides such as iridium/ruthenium oxides (IrO_2_/RuO_2_) set the benchmark for OER electrocatalysts [20–22]. However, the scarcity, prohibitive cost, and poor long-term durability of these materials restrict their wide application [23, 24]. Therefore, it is highly desirable to develop high-performance and cost-effective OER catalysts.

In this context, transition metals are considered as potential alternatives and are attracting worldwide attention due to their reasonable cost, natural abundance, high conductivity, and outstanding stability [25–29]. Among these systems, nickel-based materials show a promising potential and have been extensively studied as efficient electrocatalysts for the water oxidation reaction. Nevertheless, nickel-based composites in bulk form are not competitive for electrocatalytic applications, owing to their low specific surface area and lack of exposed reactive sites. Hence, appropriate strategies, including compositing with conductive matrices or supports, rational structure design, and doping with heteroatoms, have been adopted in order to improve their electrocatalytic activity. For instance, Xu and coworkers designed nickel nanoparticles encapsulated in N-doped graphene (denoted as Ni@NC) by annealing a Ni-based metal–organic framework (MOF) and achieved an overpotential of 280 mV at the current density of 10 mA cm^−2^, with a small Tafel slope of 45 mV dec^−1^ [30]. Liu et al. [31] fabricated nickel nanoparticles encapsulated in N-doped carbon nanotubes (Ni/N–CNTs) exhibiting an overpotential of 590 mV at 10 mA cm^−2^ and a Tafel slope of 138 mV dec^−1^, as well as high OER durability. Despite the enhanced electrochemical performance, the above materials still suffer from poor active site exposure and limited contact with electrolyte due to annealing-induced aggregation at high temperatures and structural collapse during rapid evolution of oxygen gas, resulting in significant performance degradation [32]. At variance with powders and substrate-assisted materials, self-supported binder-free metal electrocatalysts can be directly used as electrodes with increased exposure of active sites and improved electrical conductivity; this avoids the use of binders and additives while enabling full utilization of the electrode–electrolyte interface, leading to remarkable catalytic performance. More recently, nano/microstructured sponge Ni with high electrical conductivity has emerged as a novel self-supported metal network, but no OER applications have been reported.

In the present work, we report for the first time N-doped sponge nickel (denoted as N-SN), composed of interconnected Ni micro/nanofibers, as a binder-free high-efficiency OER catalysts; the N-SN material was prepared by a hydrothermal method followed by annealing in NH_3_ and exhibits a unique 3D porous structure and high electronic conductivity. The as-prepared N-SN micro/nanofibers have an open porous framework and consist of secondary nanosheets, which can significantly increase the surface area accessible to the electrolyte and expose a higher number of active sites. Due to the N-doping strategy and favorable conductive sponge architecture, the N-SN catalyst displays a remarkable OER performance, with a relatively low overpotential of 365 mV (vs. reversible hydrogen electrode, RHE) at 100 mA cm^−2^, a Tafel slope of 33 mV dec^−1^, and high stability. Further analyses, including X-ray photoelectron spectroscopy (XPS) and near-edge X-ray adsorption fine structure (NEXAFS) tests, were used to investigate the electrocatalytic OER mechanism of N-SN. Our study can draw considerable attention on substrate-free metal materials as high-performance electrocatalysts.

## Experimental

### Material Synthesis

In a typical synthesis, 1 g nickel acetate (Ni(Ac)_2_) was first dissolved in 100 mL deionized water and then, 10 mL hydrazine hydrate was added dropwise to the resulting aqueous solution. After stirring for 30 min, the above solution was transferred into a Teflon-lined steel autoclave, which was kept at 150 °C for 12 h. After naturally cooling and drying overnight, sponge Ni was obtained successfully. Then, the as-prepared sponge Ni was annealed at 300 °C for 2 h under NH_3_ (50 sccm) atmosphere to obtain N-SN. For comparison, N-doped Ni foam (N-NF) was synthesized by following a similar procedure to that used to prepare N-SN, except that Ni foam (NF) was used as the skeleton. Commercial nickel foam (1.0 × 1.0 cm^2^) was ultrasonically cleaned before use in hydrochloric acid (1 mol L^−1^), ethanol, and deionized water.

### Material Characterization

The morphologies and microstructures of the samples were characterized by field-emission scanning electron microscopy (FESEM, SU8010) and high-resolution transmission electron microscopy (HRTEM, JEM 2100F). X-ray diffraction (XRD) patterns were collected on a Rigaku D/Max-2550 instrument with Cu K_α_ radiation. XPS measurements were performed using an ESCALAB 250Xi spectrometer with an Al *K*_α_ source. Brunauer–Emmett–Teller (BET) surface area distributions were obtained with a pore size analyzer (JW-BK112). Ni L-edge NEXAFS spectra were measured at the photoemission end station of beamline BL10B of the National Synchrotron Radiation Laboratory (NSRL) in Hefei, China. A bending magnet was connected to the beamline, equipped with three gratings covering photon energies from 100 to 1000 eV. In this experiment, the samples were kept in the total electron yield mode under an ultrahigh vacuum at 5 × 10^−10^ mbar. The resolving power of the grating was typically *E*/∆*E* = 1000, and the photon flux was 1 × 10^−10^ photons s^−1^. Spectra were collected at energies from 831.4 to 884.6 eV in 0.2 eV energy steps.

### Electrochemical Measurements

The OER performances of all samples were tested in a typical three-electrode configuration using an electrochemical workstation (CH Instrument 660D). The synthesized samples (1.0 × 1.0 cm^2^) were used as the working electrode, while a standard Hg/HgO electrode and a Pt foil were used as the reference and counter electrode, respectively. The electrolyte was a 1 M KOH aqueous solution. Potentials were referenced to the RHE by adding 0.9254 V. Twenty cyclic voltammetry (CV) cycles were performed to obtain a steady current. Then, linear sweep voltammetry (LSV) curves were obtained at the scan rate of 5 mV s^−1^ in the potential range from 0.2 to 1.2 V versus Hg/HgO electrode. Tafel slopes were derived from the LSV curves. Moreover, electrochemical impedance spectroscopy (EIS) measurements were performed at the same polarization voltage for each sample, with a current density of around 10 mA cm^−2^ and within the frequency range from 0.01 Hz to 100 kHz. In order to evaluate the stability of the samples, long-term chronopotentiometry measurements were continuously conducted for 24 h at a constant current density of 10 mA cm^−2^.

## Results and Discussion

**Fig. 1:**
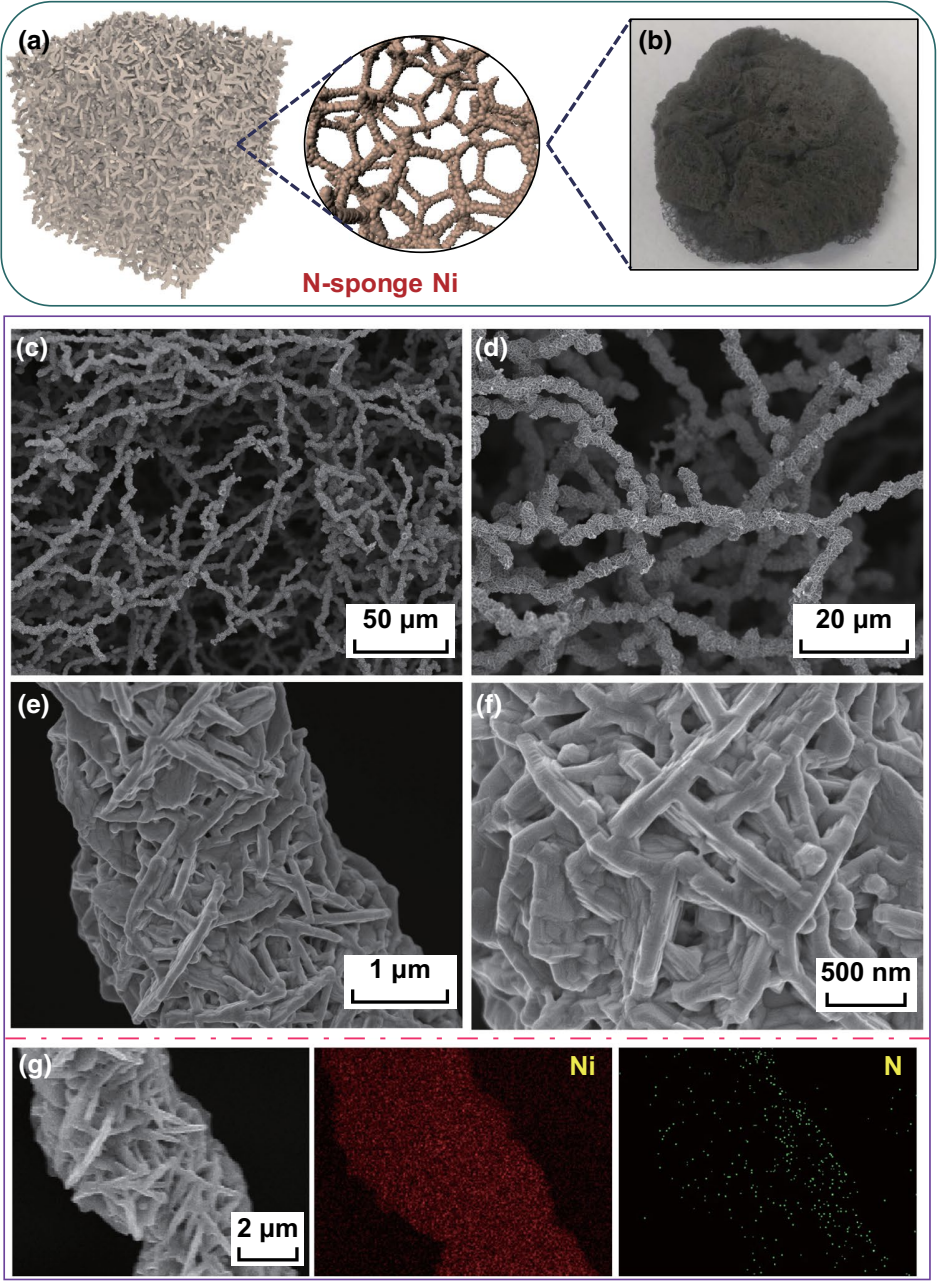
**a** Schematic illustration of N-SN. **b** Optical image of N-SN. **c**–**f** SEM images of N-SN. **g** EDS elemental mapping images of Ni and N in N-SN

Figure 1 illustrates the morphology and element distribution of the as-prepared N-SN, obtained by the combination of hydrothermal synthesis and thermal NH_3_ treatment. As shown in Fig. 1a, b, the as-obtained N-SN is a soft and porous material with a freestanding structure and good mechanical stability. It can be randomly trimmed and directly used as a binder-free electrode for OER. During the hydrothermal process, nanoplates of the precursor nickel complex are formed first; then, they are directly reduced to nickel particles with the assistance of OH^−^ ions in solution and hydrazine molecules in the nickel nanoplates. It is believed that the Ni^2+^ cations in solution are reduced on the surface of the particles, which are then joined end-to-end to form sponge Ni. SEM images of N-SN are shown in Fig. 1c–f. The interconnected N-SN micro/nanofibers form a 3D porous architecture and leave large empty spaces, which are favorable for fast ion/electron transport. Interestingly, the N-SN micro/nanofibers show a hierarchical structure consisting of cross-linked nanosheets with thicknesses of 50–150 nm. It should be noted that there are little differences in the morphologies of N-SN and sponge nickel (Fig. S1a). Both samples possess a hierarchical rough surface and porous structure. Compared with the NF and N-NF samples, possessing a smooth surface (Fig. S2a, b), the N-SN sample with smaller micro/nanofibers and rough nanosheet surfaces shows a larger surface area and could supply a higher number of active sites in the electrochemical reaction. Moreover, N-doped nickel foam (which was also fabricated by NH_3_ treatment) shows almost the same smooth texture morphology as NF (Fig. S2b), revealing that the NH_3_ treatment does not alter the morphology. In addition, energy-dispersive spectroscopy (EDS) elemental mapping images (Fig. 1g) reveal the presence and uniform distribution of Ni and N in N-SN, confirming that N was successfully doped in the as-prepared sponge Ni.

BET measurements were carried out to examine the porous nature and determine the surface area of the samples. The N_2_ adsorption/desorption isotherm curves are shown in Fig. S3. The as-prepared N-SN electrode exhibits a specific surface area of 44.4 m^2^ g^−1^, much larger than that of SN (23.7 m^2^ g^−1^), N-NF (13.6 m^2^ g^−1^), and NF (6.2 m^2^ g^−1^), indicating that the N doping and sponge structure result in larger surface areas and are beneficial for exposing a higher number of active sites, resulting in improved utilization of the active materials.

**Fig. 2:**
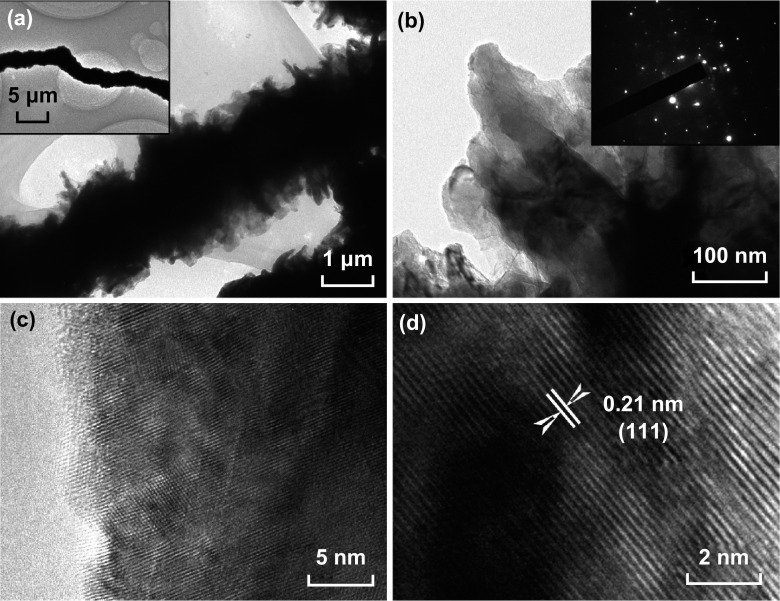
**a**–**d** TEM and HRTEM images of N-SN. The inset images in (**a**, **b**) show a low-magnification TEM image and the SAED pattern

Further insights into the microstructure of the N-SN arrays are provided by the transmission electron microscopy (TEM) analysis. Figure 2a presents a typical low-magnification TEM image of a single N-SN fiber, revealing a diameter of 1.5–2 μm. Each fiber is composed of cross-linked nanosheets (Fig. 2b) as secondary building blocks (Fig. 2c). The HRTEM image of N-SN (Fig. 2d) shows a lattice fringe of ~ 0.21 nm, corresponding to the (111) planes of Ni (JCPDS No. 04-0850). Compared to the TEM image of SN (Fig. S1b), there are no distinct changes in the appearance of each N-SN fiber. The corresponding diffraction rings in the selected-area electron diffraction (SAED) pattern confirm the nickel phase (JCPDS No. 04-0850) of N-SN.

**Fig. 3:**
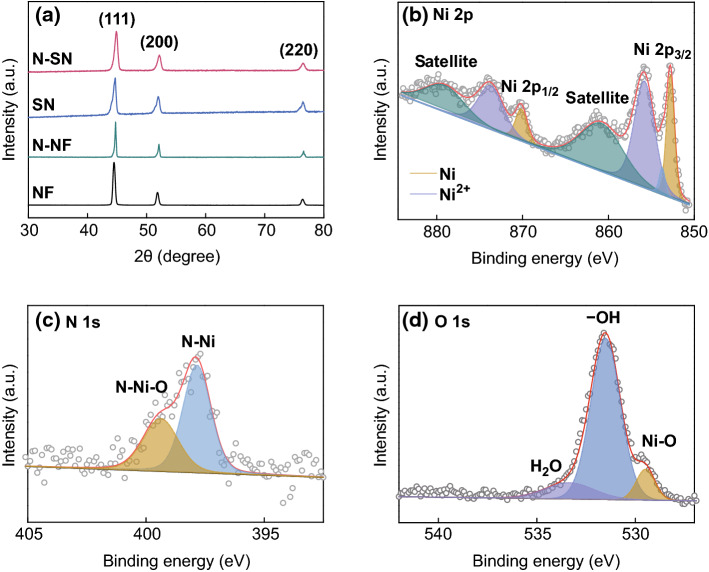
**a** XRD patterns of NF, N-NF, SN, and N-SN. XPS spectra of initial N-SN before cycling: **b** Ni 2p, **c** N 1s, **d** O 1s

The crystallographic structure and composition of the integrated N-SN samples were further characterized by XRD and XPS (Fig. 3). All identified peaks at 44.5°, 51.8°, and 76.4° can be indexed to the cubic nickel phase (JCPDS No. 04-0850, Fig. 3a). Compared to nickel foam, peak broadening (with increased full width at half maximum, FWHM) is observed for sponge nickel. According to the Scherrer formula, the peak broadening can be attributed to a reduced particle size. In addition, XPS measurements were employed to investigate the elemental composition of N-SN. In the Ni spectra of N-SN (Fig. 3b), the peaks at 852.8 and 870.1 eV are characteristic of Ni metal [33], while the peaks at binding energies at 855.8, 853.7, and 873.7 eV are assigned to Ni^2+^, indicating the surface oxidation of N-SN. The other two satellite peaks at 860.8 and 879.1 eV are in good agreement with those of the Ni^2+^ state [34]. In the N 1s spectra, the peak at 397.8 eV corresponds to N–Ni bonds, while the N 1s peak shoulder at 399.4 eV originates from the N–Ni–O bond (Fig. 3c) [35]. Moreover, the O 1s peaks (Fig. 3d) are attributed to the surface NiO resulting from surface oxidation of N-SN. The O 1s spectra consist of three main peaks and two weaker peaks at about 529.5 and 533.3 eV, which are attributed to lattice oxygen and adsorbed water, respectively. The most intense peak at 531.5 eV is due to surface hydroxyl groups [34].

**Fig. 4:**
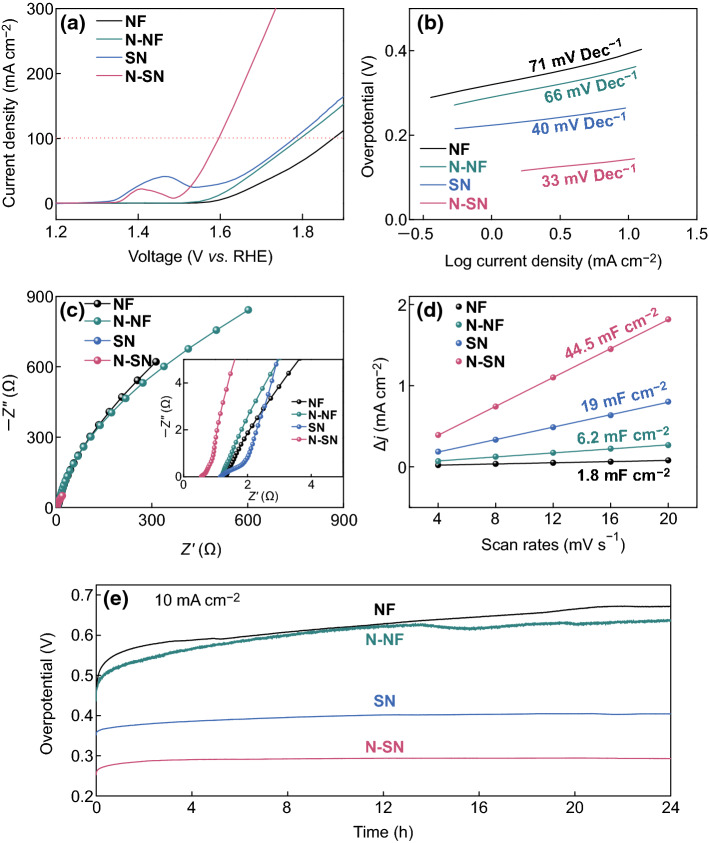
OER performance characterization and comparison of NF, N-NF, SN, and N-SN. **a** LSV curves, **b** Tafel slopes, **c** Nyquist plots, **d** current densities obtained at different scan rates, **e** electrocatalytic stability at a current density of 10 mA cm^−2^

The electrochemical OER properties of the four samples were investigated and thoroughly compared. The OER activities of NF, N-NF, SN, and N-SN were examined through LSV measurements at a scan rate of 5 mV s^−1^ (Fig. 4a). In our case, the comparison of the overpotentials of the different samples at 10 mA cm^−2^ would not be accurate, due to the presence of strong redox peaks; hence, we selected the overpotentials at 100 mA cm^−2^ for the comparison. As shown in Fig. 4a, N-SN presents an extremely low overpotential of 365 mV (vs. RHE) at a current density of 100 mA cm^−2^, lower than that of SN (549 mV), N-NF (565 mV), and NF (645 mV). Clearly, SN exhibits better OER performance than NF, implying that the design of the micro/nanostructure helps obtaining a higher number of active sites and a faster ion/electron transfer path, which accelerate the water oxidation process. The OER performances of N-SN and N-NF exhibit a significant improvement compared to those of the unmodified SN and NF, respectively; this can be ascribed to the introduction of N heteroatoms, which improves the electronic conductivity [1, 36, 37]. The superior OER catalytic performance of N-SN can be ascribed to the combination of its micro/nanoscale structure and enhanced electrical conductivity. The Tafel slopes in Fig. 4b were used to investigate the OER kinetics. The Tafel equation *η *= *a *+ *b*log *j* (where *η*, *j*, *b,* and *a* represent the overpotential, current density, Tafel slope, and a constant depending on the electrode materials, respectively) can be applied in the high-polarization region. At low polarization values, the dependence of the current on the polarization is usually linear, rather than logarithmic. As a result, the current range between 1 and 10 mA cm^−2^ is explored. A smaller Tafel slope denotes a faster OER kinetics [38], because of the faster increase in current densities with increasing overpotentials. The Tafel slope of SN is approximately 40 mV dec^−1^, which further decreases to 33 mV dec^−1^ for N-SN, while the corresponding values of N-NF and NF reach 66 and 71 mV dec^−1^, respectively. The lowest Tafel slope of N-SN denotes its fastest kinetics [39–41], which was further confirmed by EIS measurements. Figure 4c shows the Nyquist plots of the four samples at the overpotential of 195 mV. In the high-frequency region, the *x*-axis intercept of N-SN is smaller than that of SN, N-NF, and NF, suggesting that the micro/nanostructures of SN show a smaller resistance, and that N doping is beneficial for further enhancing the electronic conductivity. This reveals that the combination of micro/nanostructures and N doping in N-SN promotes faster electron transfer and ion diffusion, thus leading to better OER catalytic performances.

The electrochemical active surface areas (ECSAs) of N-SN, SN, N-NF, and NF were estimated from the electrochemical double-layer capacitance (*C*_dl_) of each catalytic surface. The plots in Fig. 4d were obtained by measuring the non-Faradaic capacitive current associated with double-layer charging from the scan rate dependence of the cyclic voltammograms (Fig. S4). The ECSA is expected to be linearly proportional to the *C*_dl_ value, which is equal to half the slope of the plot [42]. It should be pointed out that the *C*_dl_ value of N-SN is about 44.5 mF cm^−2^, about seven times higher than that of N-NF (6.2 mF cm^−2^), as well as higher than that of SN (19 mF cm^−2^) and NF (1.8 mF cm^−2^). These obvious differences indicate that the tailored design and fabrication of N-doped SN samples with micro/nanoscale structures lead to a marked increase in the number of active sites for OER, resulting in enhanced OER catalytic properties. On the other hand, the *C*_dl_ values of N-SN and N-NF are much higher than those of SN and NF, respectively, indicating that the N-doping strategy also plays an important role in increasing the electrochemical active surface area. It can be thus be concluded that the combination of N doping and micro/nanostructure design takes full advantage of the available electrochemical active sites, improving the catalytic performance of the samples. The turnover frequency (TOF) is regarded as the best parameter to compare the intrinsic activities of electrocatalysts at various loadings. Assuming that all metal atoms in the samples are active and accessible to the electrolyte, the TOF values can be obtained according to the equation TOF = *iN*_A_/(4*FN*_atoms_) where *i*, *N*_A_, *F*, and *N*_atoms_ represent the current density at a specific overpotential, the Avogadro constant, the Faraday constant, and the number of atoms or active sites, respectively [43, 44]. The TOF of N-SN at the overpotential of 400 mV is calculated to be 1.190 s^−1^, which is much higher than that of SN (0.290 s^−1^), N-NF (0.137 s^−1^), and NF (0.065 s^−1^), indicating a better intrinsic activity of N-SN. In addition, the Faradaic efficiency was obtained by comparing the measured gas volume with the theoretical one. The Faradaic efficiency of N-SN is about 100%, because of the good agreement between the experimental and calculated volumes of evolved O_2_ (Fig. S5).

**Fig. 5:**
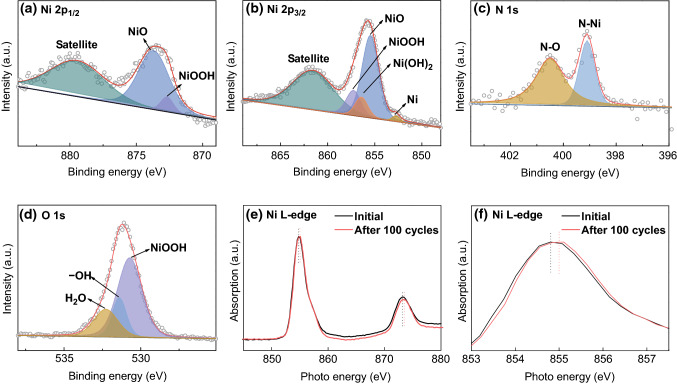
XPS spectra of N-SN after 100 cycles. **a** Ni 2p_1/2_, **b** Ni 2p_3/2_, **c** N 1s, **d** O 1s. **e**–**f** NEXAFS spectra of N-SN before and after 100 OER cycles

To access and compare the OER stability of our four samples, long-term chronopotentiometry measurements were conducted at 10 mA cm^−2^ for 24 h (Fig. 5e). The curve obtained for NF presents a clear increasing trend, demonstrating a higher overpotential and worse durability in the OER process. The curve corresponding to N-NF displays large fluctuations in the later stages of the reaction, suggesting unsatisfactory durability. In contrast, the curves obtained for SN and N-SN remain flat and no noticeable overpotential increases are observed, indicating that N-SN and SN retain a steady OER activity. In particular, the fluctuations in the N-SN curve remain in the range of 10 mV, which demonstrates the excellent stability of N-SN. The overpotential at 10 mA cm^−2^ of N-SN is much smaller than that of SN, N-NF, and NF. The SEM images of N-SN after 100 cycles (Fig. S5) confirm that the interconnected structures are preserved well, without collapse or breakage. The outstanding mechanical stability of N-SN is beneficial for retaining a good electrochemical durability in alkaline aqueous solution.

To further illustrate the OER mechanism of N-SN, XPS and NEXAFS were used to examine the samples after 100 cycles and identify the actual active materials on the surface of N-SN (Fig. 5). According to the XPS analysis, the active peaks before and after the OER cycles change considerably, indicating that pure N-SN is not the active surface materials for OER and new products are produced during the OER process. The Ni 2p spectra (Fig. 5a, b) show a marked decrease in the intensity of the peak at 852.8 eV, characteristic of Ni metal, while the previous peak at 870.1 eV disappears. At the same time, the existence of Ni^3+^ species is evidenced by the peaks at 857.2 and 872.7 eV, assigned to γ-NiOOH originating from the surface oxidation of N-SN, which proceeds as follows: Ni^2+^ + 3OH^−^ − 3e^−^ → NiOOH + H_2_O [45]. γ-NiOOH is probably the active species actually contributing to the OER process. The binding energy at 856.4 eV corresponds to Ni(OH)_2_, because γ-NiOOH is not stable and will transform into Ni(OH)_2_ as follows: NiOOH + e^−^ + H_2_O → Ni(OH)_2_ + OH^−^ [46]. Moreover, the peaks at 855.4 and 873.7 eV, attributed to NiO, remain almost unchanged because of the surface oxidation of N-NS. Figure 5c shows peaks at 399.0 and 400.5 eV corresponding to N–Ni and N–O bonds, respectively. The separation and shift of the N 1s region reflect a change in chemical conditions after 100 cycles. A clear shift in the main O 1s peaks is observed in Fig. 5d. The peak located at 530 eV is characteristic of γ-NiOOH, which is the typical species consistent with lattice oxygen [45]. This clearly demonstrates the existence of γ-NiOOH, consistent with the Ni 2p spectra discussed above. The two remaining peaks at 531.4 and 532.3 eV correspond to surface hydroxyl groups and adsorbed water, respectively. As shown in Fig. 5e, f, the L-edge NEXAFS results show the local structural variation around Ni sites in N-SN. The L-edge NEXAFS technique is one of the best tools to investigate the electronic structure of first-row transition metals. The initial N-SN shows a sharp L_3_ maximum near 854.7 eV and a relatively broad L_2_ edge, while a primary L_3_ peak near 855.0 eV and a relatively similar L_2_ region are observed after 100 cycles. Generally, the average L_3_ absorption centroid shifts to higher energies as Ni is oxidized from Ni^I^ to Ni^II^, Ni^III^, and Ni^IV^. In other words, the L_3_ peak of N-SN shifts ~ 0.3 eV higher after 100 cycles, which indicates that Ni is oxidized to higher valence states during the OER process. In addition, as shown in the SEM images of N-SN after 100 cycles (Figs. S6, S7), the morphology of N-SN changes significantly and new small nanosheets are formed on the pristine secondary nanosheets due to the growth of γ-NiOOH and Ni(OH)_2_, in agreement with the XPS and NEXAFS analyses discussed above.

## Conclusion

We have rationally designed and fabricated N-doped sponge nickel as a novel and efficient OER electrocatalyst. Using a hydrothermal method combined with a thermal treatment in NH_3_ atmosphere, a self-supported N-SN consisting of micro/nanostructured fibers was successfully synthesized. The new material exhibits a 3D porous sponge skeleton with increased accessible surface area. The N-SN electrode shows high conductivity, large surface area, and abundant active sites, which result in excellent electrocatalytic performance, with low overpotential and high cycling stability. XPS and NEXAFS measurements were used to study the OER mechanism of N-SN; γ-NiOOH, originating from the oxidation of N-SN in alkaline solution, is identified as the actual active material for OER. In this work, we have not only demonstrated the potential of N-SN as a novel electrocatalyst, but also provided insights into how the sponge structure and N-doping strategy can enhance the electrocatalytic performance.

## Electronic supplementary material

Below is the link to the electronic supplementary material.

Supplementary material 1 (PDF 589 kb)
