# Supplementary material for: Nitrogen-Doped Sponge Ni Fibers as Highly Efficient Electrocatalysts for Oxygen Evolution Reaction
Source: Nanomicro Lett. 2019 Mar 9;11:21. doi: 10.1007/s40820-019-0253-5 (PMC7770937; doi:10.1007/s40820-019-0253-5)
Supplement: Supplementary file 1 — Supplementary material 1 (PDF 589 kb) [file 40820_2019_253_MOESM1_ESM.pdf]

Supporting Information for

Nitrogen-Doped Sponge Ni Fibers as Highly Efficient Electrocatalysts for Oxygen Evolution Reaction

Kaili Zhang[1], Xinhui Xia[1, *], Shengjue Deng[1], Yu Zhong[1], Dong Xie[2], Guoxiang Pan[3], Jianbo Wu[4], Qi Liu[5], Xiuli Wang[1], Jiangping Tu[1, *]

[1]State Key Laboratory of Silicon Materials, Key Laboratory of Advanced Materials and Applications for Batteries of Zhejiang Province, and Department of Materials Science and Engineering, Zhejiang University, Hangzhou 310027, People's Republic of China

[2]Guangdong Engineering and Technology Research Center for Advanced Nanomaterials, School of Environment and Civil Engineering, Dongguan University of Technology, Dongguan 523808, People's Republic of China

[3]Department of Materials Chemistry, Huzhou University, Huzhou 313000, People's Republic of China

[4]Zhejiang Provincial Key Laboratory for Cutting Tools, Taizhou University, Taizhou 318000, People's Republic of China

[5]Department of Physics, City University of Hong Kong, Kowloon 999077, Hong Kong

*Corresponding authors. E-mail: helloxxh@zju.edu.cn (Xinhui Xia), tujp@zju.edu.cn (Jiangping Tu)

Supplementary Figures

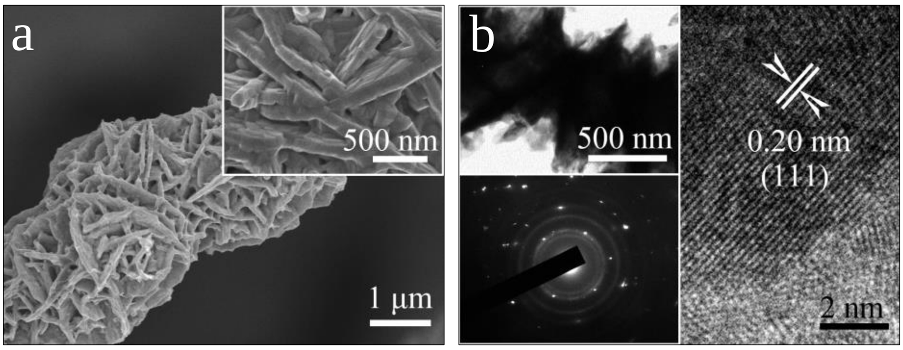

Fig. S1 **a** SEM image and **b** TEM image of SN

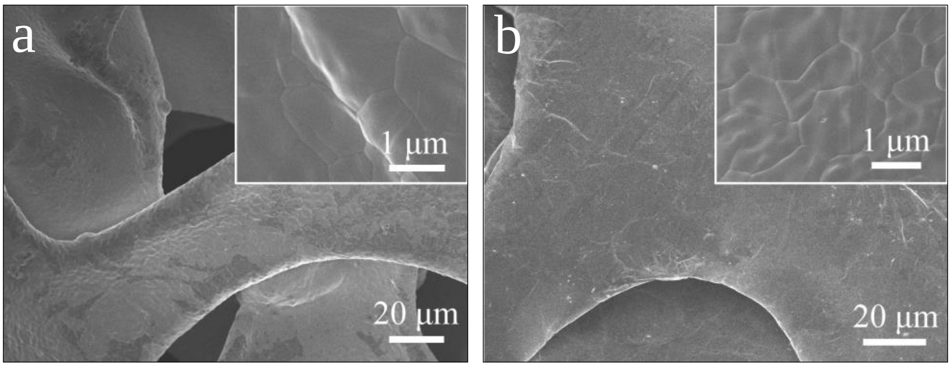

Fig. S2 SEM images of **a** NF, **b** N-NF

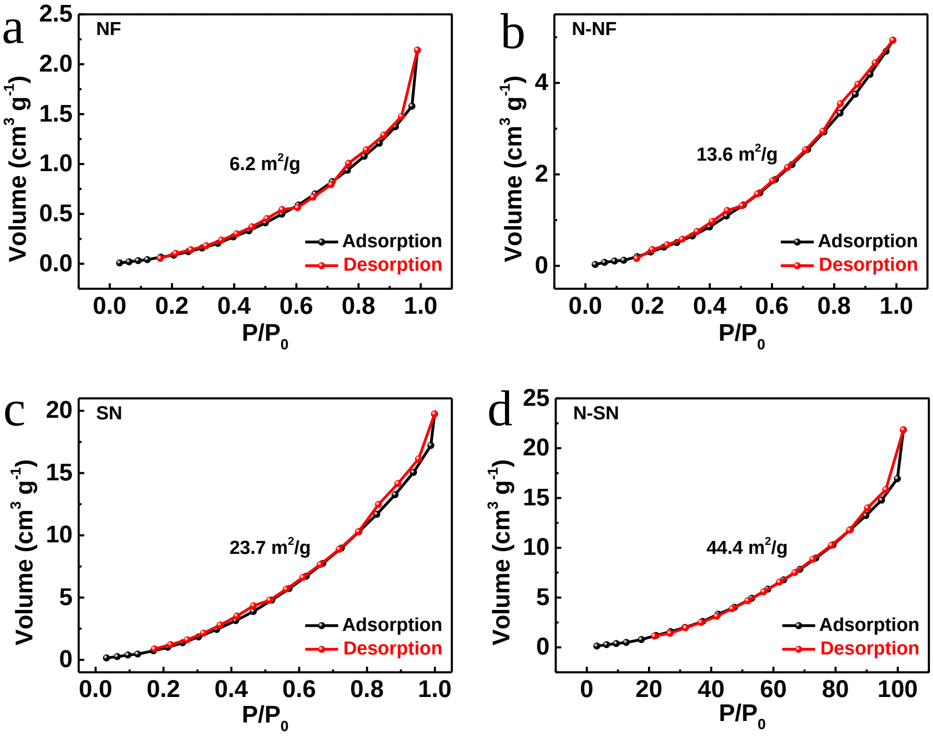

Fig. S3 BET measurements: nitrogen adsorption-desorption isotherm curves of **a** NF, **b** N-NF, **c** SN and **d** N-SN

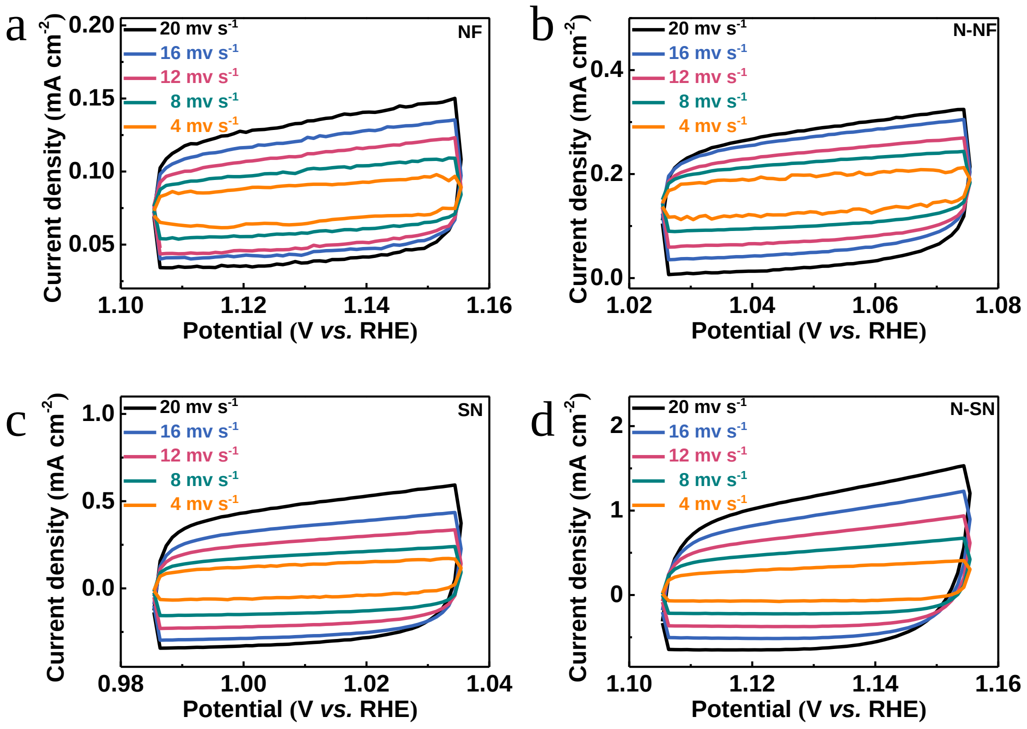

Fig. S4 CV curves of different samples in double layer region at scan rates of 4, 8, 12, 16, and 20 mV s^{-1}: **a** NF, **b** N-NF, **c** SN, and **d** N-SN

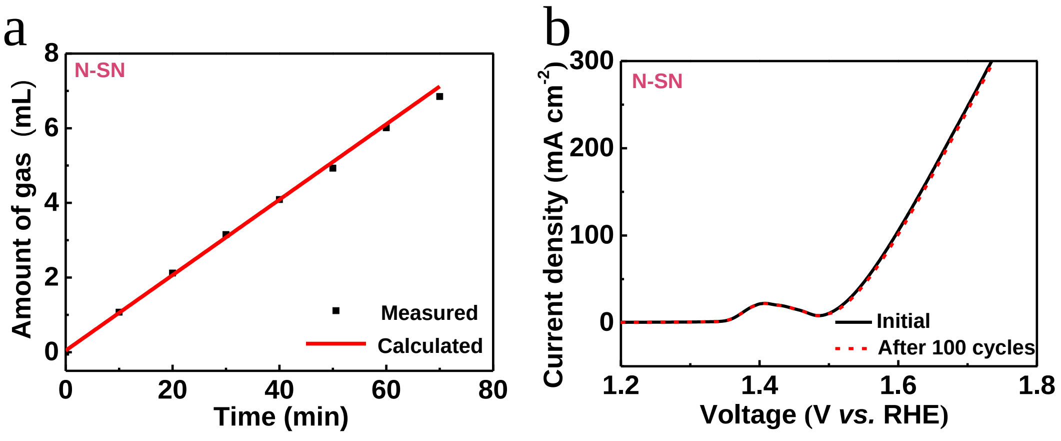

Fig. S5 a Faradic efficiency: comparison between the theoretically calculated amount of O_2 and experimentally measured O_2 value versus reaction time at a constant current density of 10 mA cm^{-2}. **b** LSV comparison of N-SN between initial state and after 100 cycles

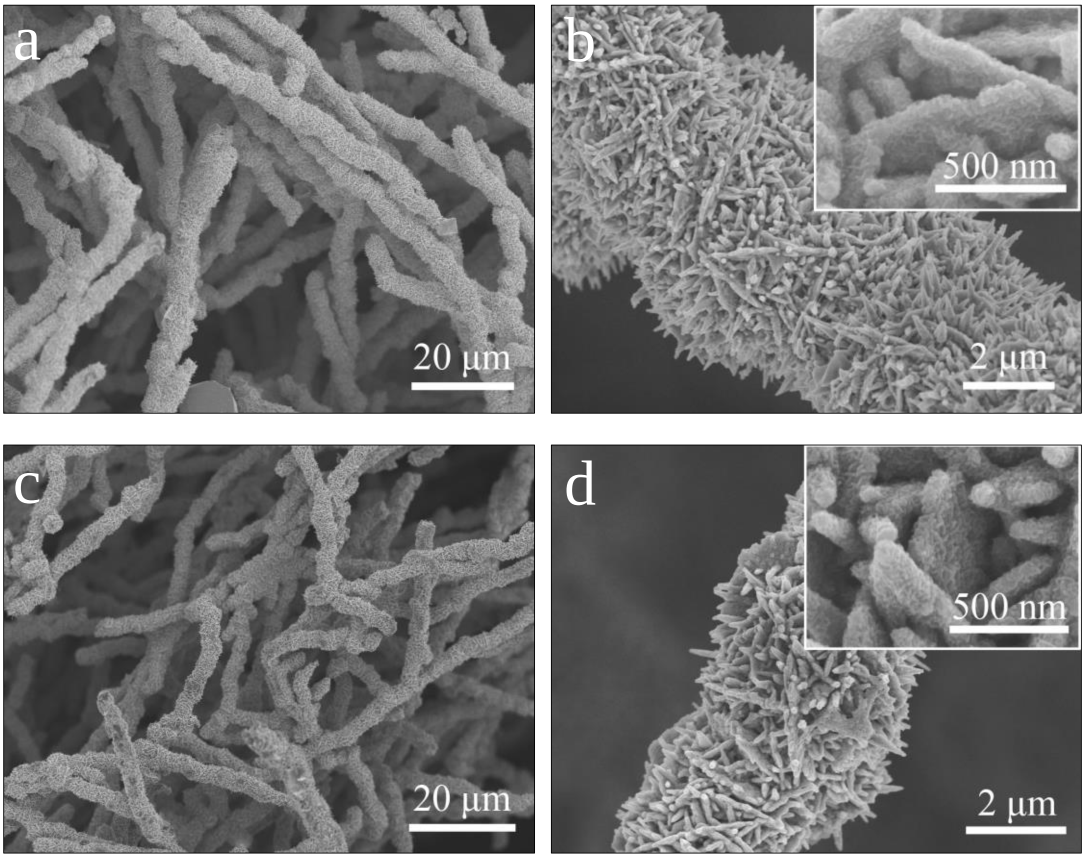

Fig. S6 SEM images of **a, b** SN, **c, d** N-SN after 100 OER cycles

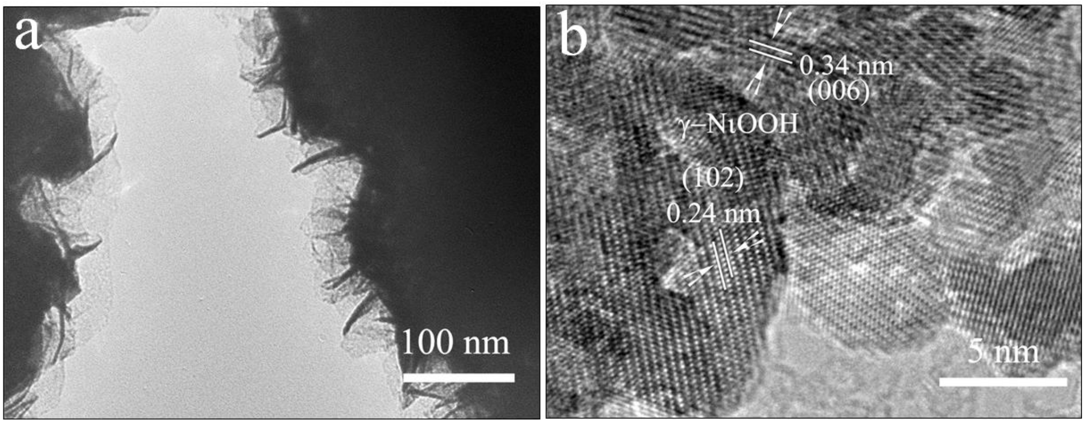

Fig. S7 TEM images of N-SN after 100 OER cycles
